# Poultry fat decreased fatty acid transporter protein mRNA expression and affected fatty acid composition in chickens

**DOI:** 10.1186/2049-1891-3-17

**Published:** 2012-05-31

**Authors:** Jianmin Yuan, Bingkun Zhang, Yuming Guo

**Affiliations:** 1 State Key Laboratory of Animal Nutrition, College of Animal Science and Technology, China Agricultural University, Beijing, 100193, P. R. China

**Keywords:** Breast muscle, FATP mRNA, Fat type, Intestine, L-FABP mRNA

## Abstract

**Background:**

A study was undertaken to examine the effects of poultry fat (PF) compared with those of soybean oil (SBO) on intestinal development, fatty acid transporter protein (FATP) mRNA expression, and fatty acid composition in broiler chickens. A total of 144 day-old male commercial broilers were randomly allocated to 2 treatment groups (6 replicates of 12 chicks for each treatment) and fed isocaloric diets containing 3.0% PF or 2.7% SBO at 0 to 3 wk and 3.8% PF or 3.5% SBO at 4 to 6 wk, respectively.

**Results:**

PF had no influence on intestinal morphology, weight, or DNA, RNA, or protein concentrations at 2, 4, and 6 wk of age. However, compared with SBO, PF significantly decreased FATP mRNA abundance at 4 wk (*P* = 0.009) and 6 wk of age (*P* < 0.001); decreased liver fatty acid-binding protein (L-FABP) mRNA abundance at 6 wk of age (*P* = 0.039); and decreased C18:2 (*P* = 0.015), C18:3 (*P* < 0.001), C20:2 (*P* = 0.018), Σ-polyunsaturated fatty acids **(**Σ-PUFA) (*P* = 0.020), and the proportion of PUFA (*P* < 0.001) in the intestinal mucosa and decreased C18:2 (*P* = 0.010), C18:3 (*P* < 0.001), C20:2 (*P* < 0.001), Σ-PUFA (*P* = 0.005), and the proportion of PUFA (*P* < 0.001) in breast muscle at 6 wk of age.

**Conclusions:**

PF decreases FATP and L-FABP mRNA expression and decreased the proportion of PUFA in the intestinal mucosa and breast muscle.

## Background

As an organ of nutrient absorption, the small intestine is very important for animals. The capacity to absorb nutrients depends mainly on the development of the intestinal mucosa. A previous study showed that morphological characteristics of the small intestinal mucosa are affected by diet [1]. Dietary lipids could affect intestinal morphology [2-4], influencing the fatty acid (FA) composition of the apical enterocyte membrane [5] and the fluidity of brush border membranes [6]. This in turn could alter the transportation and diffusion of certain nutrients across the intestine [7].

Poultry fat (PF), an available ingredient source, has been widely used as an energy source in swine feeds. A previous study showed that PF appeared to be more efficiently utilized than swine fat for both body weight (BW) gain and the production of carcass energy [8] and did not influence swine performance compared with soybean oil (SBO) [9]. However, few data exist on the effect of PF on the development of intestinal structure and on the absorption and utilization of FA compared with other animal fats or plant oil.

Recently, there has been an increasing interest in improving the quality of meat. Flavor, which is easily perceived by the consumer, is an important evaluation factor of meat quality. Previous studies showed that dietary fat could affect the FA composition in animals. PF increased linoleic acid and decreased oleic acid contents in the longissimus muscle of pigs compared with beef tallow [10]. Apple et al. [9] showed that PF decreased the polyunsaturated FA (PUFA) content and PUFA to saturated FA (SFA) ratio in the longissimus muscle of pigs compared with SBO. Another previous study showed that the FA composition in breast muscle of broilers was associated with liver FA-binding protein (L-FABP) mRNA expression in intestinal mucosa [11], and FABP expression in the small intestine could be regulated by FA [12]. However, little attention has been given to the effect of PF on the FA composition and function of the intestines in broilers. The aim of the present study was to evaluate the effects of PF on the development of intestinal structure, fatty acid transfer protein (FATP) mRNA expression in the small intestine, and FA composition in the intestinal mucosa and breast muscle of broiler chickens compared with SBO.

## Methods

### Experimental design, animals, and diets

A total of 144 day-old Arbor Acres Plus male commercial broilers obtained from a commercial hatchery were randomly allocated to 12 pens. Each treatment was assigned to 6 replicates of 12 chicks, and broilers were fed a 2-phase diet with transition from starter (0 to 3 wk) to grower (4 to 6 wk). Starter diets were isoenergetically supplemented with 3.0% PF or 2.7% SBO, and grower diets were isoenergetically supplemented with 3.8% PF or 3.5% SBO. Thus, the corn/soybean meal content varied between diets. The nutrient analyzed values met or exceeded the recommendations of NY/T33-2004. The composition of the experimental diet and FA compositions of the diets are presented in Table 1.

**Table 1:** **Composition**^**1**^**of the experimental diet (g/100 g of diet)**

Ingredients	Starter (0-3 wk)		Grower (4-6 wk)	
	Poultry fat	Soybean oil	Poultry fat	Soybean oil
Corn	54.75	55.12	59.10	59.50
Soybean meal	38.17	38.10	33.50	33.40
Limestone	1.20	1.20	1.20	1.20
Dicalcium phosphate	1.90	1.90	1.60	1.60
Salt	0.35	0.35	0.35	0.35
Soybean oil	0.00	2.70	0.00	3.50
Poultry fat	3.00	0.00	3.80	0.00
98% DL-methionine	0.18	0.18	0.11	0.11
4% flavomycin	0.04	0.04	0.02	0.02
33% ethoxyquin	0.03	0.03	0.00	0.00
Trace mineral premix^3^	0.20	0.20	0.20	0.20
Vitamin Premix^4^	0.02	0.02	0.02	0.02
50% choline chloride	0.16	0.16	0.10	0.10
Calculated analysis^5^				
AME, kcal/kg	2,910	2,910	3,000	3,000
Crude protein,%	20.68	20.68	19.00	19.00
Lysine,%	1.12	1.12	1.00	1.00
Methionine + cysteine,%	0.83	0.83	0.71	0.71
Calcium,%	0.98	0.98	0.90	0.90
Available phosphorus,%	0.45	0.45	0.40	0.40
Determined analysis				
C14:0, mg/g	0.13	0.04	0.19	0.05
C14:1, mg/g	0.02	−^2^	0.05	-
C16:0, mg/g	10.14	6.35	12.83	7.33
C16:1, mg/g	1.47	0.10	1.98	0.16
C18:0, mg/g	2.12	1.58	2.81	1.91
C18:1, mg/g	16.13	10.22	19.88	12.48
C18:2, mg/g	18.88	23.87	20.67	28.67
C18:3, mg/g	1.07	2.12	1.12	2.60
C20:0, mg/g	0.13	0.20	0.17	0.23
C20:1, mg/g	0.19	0.15	0.25	0.19
C20:2, mg/g	0.05	0.02	0.06	0.02
C20:3, mg/g	0.04	-	0.05	-
C22:0, mg/g	0.17	0.53	0.17	0.32
C22:1, mg/g	0.06	0.14	0.11	0.17
C24:0, mg/g	0.03	1.61	0.10	2.45
SFA, mg/g	12.72	10.31	16.27	11.06
MUFA, mg/g	17.87	10.61	22.27	13.00
PUFA, mg/g	20.04	26.03	21.90	31.29
Total FA, mg/g	50.63	46.95	60.44	55.35
SFA, %	25.12	21.96	26.92	19.98
MUFA, %	35.30	22.60	36.85	23.49
PUFA, %	39.58	55.44	36.23	56.53
MUFA/SFA	1.41	1.03	1.37	1.18
PUFA/SFA	1.58	2.52	1.35	2.83

^1^Data are expressed on an as-fed basis.

^2^Undetermined.

^3^Trace mineral premix provided per kilogram of diet: Fe, 80 mg (as FeSO_4_·H_2_O); Cu, 8 mg (as CuSO_4_·5H_2_O); Zn, 100 mg (as ZnSO_4_·H_2_O); Mn, 100 mg (as MnSO_4_·H_2_O); Se, 0.3 mg (as Na_2_SeO_3_); I, 0.50 mg (as Ca(IO_3_)_2_.

^4^Vitamin premix provided per kilogram of diet: Vitamin A (as retinyl acetate), 12,500 IU; cholecalciferol, 2,500 IU; vitamin E (as dl-α-tocopherol acetate), 18 IU; menadione, 3 mg; thiamine, 2.5 mg; riboflavin, 6.6 mg; pyridoxine, 4.9 mg; pantothenic acid, 14.7 mg; niacin, 36.8 mg; folic acid, 1.2 mg; biotin, 0.013 mg; cobalamine, 0.025 mg.

^5^Calculated value.

All birds were housed in the same experimental chicken house under 24 h continuous light. The environmental temperature was initially set at 33°C and then gradually reduced to 21°C. All chickens had free access to water and the experimental diets. The present study was approved by the China Agricultural University and was carried out in accordance with the Guidelines for Experimental Animals.

### Tissue sampling and preparation

At 2, 4, and 6 wk of age, six chicks from each treatment group were anesthetized by intravenous injection of sodium 75 pentobarbitone (1 mL/kg BW) after fasting for 6 h. The intestine was removed and weighed. The jejunal mucosa near Meckel’s diverticulum was removed by gentle scraping with a clean microscope slide, washed with cold phosphate buffered saline, frozen in liquid nitrogen, and stored at −80°C for the determination of L-FABP and FATP mRNA levels and DNA, RNA, and protein concentrations. A 1 cm piece of jejunum near Meckel’s diverticulum was removed and flushed with 0.9% NaCl and then fixed in 10% neutral buffered formalin solution for morphometric analysis. Morphological observation was performed on 5 μm sections under a light microscope (Nikon Eclipse TE2000-S) according to Sklan and Noy [13].

### DNA, RNA, and protein assay

The RNA, DNA, and protein were extracted from segments frozen in liquid nitrogen using TRI Reagent RNA/DNA/Protein Isolation Reagent (Invitrogen Life Technologies, Carlsbad, CA), and mean concentrations were determined colorimetrically according to Uni et al. [14].

### Total RNA isolation and reverse transcription

Total RNA was isolated from jejunal mucosa using TRIzol Reagent (1334257; Invitrogen Life Technologies, Carlsbad, CA) according to the manufacturer’s instructions. RNA integrity was assessed via agarose gel electrophoresis, and RNA concentration and purity were determined spectrophotometrically using A260 and A280 measurements. Reverse transcription (RT) reactions (20 μL) comprised 1 μg total RNA, 20 U of an RNAse inhibitor (Promega), 10 mmol dNTPs (Sigma), 4.0 μL of 5 × M-MLV RT reaction buffer (Promega), 100 U M-MLV transcriptase (Promega), and 1.0 μL Oligo (dT) 12–18 (Promega). Cycle parameters for the RT procedure were 1 cycle at 20°C for 5 min, 1 cycle at 42°C for 60 min, and 1 cycle at 70°C for 5 min. The reaction was stopped by placement on ice. The RT products (cDNA) were stored at −20°C for relative gene abundance by PCR.

### Real-time PCR for abundance of L-FABP and FATP mRNA

Quantitative analysis of PCR was performed with the PRISM 7700 Fluorescence Detection System (ABI Biosystems) according to optimized PCR protocols and the SYBR Green qPCR kit (ABI Biosystems 4309155), in which SYBR Green was a double-stranded, DNA-specific fluorescent dye. The PCR reaction system (20 μL) contained 10 μL SYBR Green PCR Master Mix, 2.0 μL primer (1.0 μL forward and 1.0 μL reverse; see Table 2 for primer sequence), and 2.0 μL cDNA template. For the PCR reaction, the experimental protocol was as follows: denaturation program (95°C for 3 min), amplification and abundance program repeated 42 times (94°C for 30 s, 51°C for 30 s, and 72°C for 60 s with a single fluorescence measurement), and extension (72°C for 7 min). Relative standard curve methods were used to calculate the abundance of gene expression. Briefly, copy numbers were determined from two independent cDNA preparations of any sample. Copy numbers were calculated relative to a dilution series of the respective reference plasmids, comprising 103 to 108 copies. The reference plasmids contained the cloned RT-PCR products obtained with these primers. The housekeeping gene, β-actin, was used as an internal standard for the PCR reaction.

**Table 2:** Oligonucleotide PCR primers

Name	Oligo	Primer sequence	Predicted size (bp)	GenBank accession
L-FABP	Forward Reverse	5’-GAAGGGTAAGGACATCAA-3’ 5’-TCGGTCACGGATTTCAGC-3’	219	NM_204192
FATP-1	Forward Reverse	5’-GACTGCGCCAAGTACAGATGC-3’ 5’-CACTCGGTGGCTCCGTAGAAC-3’	203	DQ352834
β-actin	Forward Reverse	5’-CCACCGCAAATGCTTCTAAAC-3’ 5’-AAGACTGCTGCTGACACCTTC-3’	175	NM_205518

### Assay of FA in jejunal mucosa and breast muscle

The mucosa of the jejunum was removed by gentle scraping with a clean microscope slide at 6 wk of age, and together with breast muscle, was weighed and lyophilized. The FA compositions of jejunal mucosa, breast muscle, and diet were determined according to Sukhjja and Palmquist [15] with minor modifications. Samples containing 10 to 50 mg of lipids were accurately weighed, and 4 mL of n-hexane containing internal standard (heptadecanoic acid, C17:0, Fluka 51633) and 3 mL of freshly made 5% methanolic HCl were added and heated for 2 h in a water bath at 80°C to methylate. The FA content was determined using a gas chromatograph HP 6890 equipped with a flame ionization detector and an HP-INNOWA capillary column. Helium was used as the carrier gas. The oven temperature was programmed as follows: from 140°C to 200°C at 1.50°C/min; from 200°C to 220°C at 100°C/min; and from 220°C to 230°C at 20°C/min. The other chromatographic conditions were: injector and detector temperatures, 200°C; and injected sample volume, 1 μL. FA were identified by matching their retention times with those of their relative standards.

### Statistical analysis

The data were compared using SPSS 10.0 with an independent-samples *t*-test, and a *P*-value of <0.05 was considered statistically significant.

## Results

### Intestinal development

PF had no influence on the weight of the jejunum or the villus height, crypt depth, or villus height/crypt depth of the jejunal mucosa compared with SBO in broilers with mean BW to each age (data not shown). DNA, RNA, and protein concentrations in jejunal mucosa were comparable in PF- and SBO-fed birds.

### L-FABP and FATP mRNA abundance

PF had no influence on L-FABP (*P =* 0.79) or FATP (*P =* 0.23) mRNA abundance of jejunal mucosa at 2 wk of age, and there was no difference in L-FABP mRNA abundance at 4 wk of age (*P* = 0.26) (Table 3). However, compared with SBO, PF significantly decreased FATP mRNA abundance at 4 wk (*P =* 0.009) and 6 wk of age (*P* < 0.001) and significantly decreased L-FABP mRNA abundance at 6 wk of age (*P* = 0.039).

**Table 3:** L-FABP and FATP mRNA abundance

Age	mRNA	Poultry fat	Soybean oil	SEM	*P* value
2 wk	L-FABP	0.078	0.083	0.010	0.791
	FATP	0.32	0.40	0.034	0.233
4 wk	L-FABP	0.21	0.28	0.029	0.260
	FATP	0.58	1.66	0.211	0.009
6 wk	L-FABP	0.05	0.15	0.019	0.039
	FATP	0.38	2.33	0.339	<0.001

### FA composition of intestinal mucosa and breast muscle

In the jejunal mucosa, PF decreased the content of C18:2 (*P* = 0.015), C18:3 (*P* < 0.001), C20:2 (*P =* 0.018), and ΣPUFA (*P* < 0.020); the proportion of PUFA (*P* <0.001); and the ratio of PUFA/SFA (*P* = 0.006) compared with SBO at 6 wk of age (Table 4). However, PF significantly increased the content of C24:0 (*P* = 0.008) and the proportion (%) of SFA (*P =* 0.024) and monounsaturated FA (MUFA) (*P* < 0.001).

**Table 4:** Fatty acid concentration of intestinal mucosa and breast muscle (fresh) of broilers at 6 wk of age

Fatty acid	Intestinal mucosa				Breast muscle			
	Poultry fat	Soybean oil	SEM	*P* value	Poultry fat	Soybean oil	SEM	*P* value
C14:0, mg/g	0.11	0.08	0.013	0.193	0.04	0.03	0.006	0.385
C14:1, mg/g	0.03	0.02	0.005	0.125	0.08	0.08	0.002	0.707
C16:0, mg/g	4.85	4.31	0.511	0.535	2.19	1.93	0.128	0.348
C16:1, mg/g	0.91	0.46	0.128	0.077	0.36	0.21	0.047	0.021
C18:0, mg/g	3.58	3.73	0.243	0.488	1.03	1.05	0.038	0.833
C18:1, mg/g	6.24	4.32	0.762	0.160	3.08	2.23	0.266	0.113
C18:2, mg/g	5.86	8.24	0.636	0.015	1.83	2.60	0.164	0.010
C18:3, mg/g	0.18	0.45	0.044	<0.001	0.05	0.14	0.015	<0.001
C20:0, mg/g	0.07	0.07	0.009	0.439	0.01	0.01	0.001	1.000
C20:1, mg/g	0.10	0.09	0.013	0.746	0.05	0.03	0.004	0.029
C20:2, mg/g	0.08	0.12	0.010	0.018	0.07	0.11	0.007	<0.001
C20:3, mg/g	1.27	1.01	0.107	0.137	0.61	0.69	0.022	0.056
C22:0, mg/g	0.43	0.42	0.064	0.436	0.09	0.08	0.007	0.345
C24:0, mg/g	0.43	0.25	0.076	0.008	0.08	0.19	0.030	0.075
ΣSFA, mg/g	9.36	8.66	0.717	0.687	3.40	3.26	0.159	0.680
ΣMUFA, mg/g	7.28	4.89	0.899	0.145	3.51	2.56	0.315	0.089
ΣPUFA, mg/g	7.40	9.82	0.676	0.020	2.56	3.54	0.196	0.005
ΣFA, mg/g	24.04	23.37	1.704	0.854	9.48	9.36	0.506	0.916
SFA, %	39.27	37.16	0.814	0.024	36.15	34.89	0.455	0.124
MUFA, %	29.48	20.71	1.687	<0.001	36.62	27.21	1.669	<0.001
PUFA, %	31.25	42.13	1.040	<0.001	27.24	37.90	1.720	<0.001
MUFA/SFA	0.75	0.56	0.040	<0.001	1.02	0.78	0.042	<0.001
PUFA/SFA	0.80	1.13	0.052	0.006	0.75	1.09	0.053	<0.001

In the breast muscle of broilers at 6 wk of age, PF decreased the content of C18:2 (*P* = 0.010), C18:3 (*P* < 0.001), C20:2 (*P* < 0.001), and ΣPUFA (*P =* 0.005); the proportion (%) of PUFA (*P* < 0.001); and the ratio of PUFA/SFA (*P* < 0.001). However, PF increased the content of C16:1 (*P* = 0.021) and C20:1 (*P* = 0.029); the proportion (%) of MUFA (*P* < 0.001); and the ratio of MUFA/SFA (*P* < 0.001).

## Discussion

In the current study, PF supplementation had no influence on the weight of the jejunum, villus height, crypt depth, or villus height/crypt depth compared with SBO in broilers of different ages. A previous study in rodents showed that different sources of dietary fat had different effects on the intestinal morphology of rats. Corn oil and olive oil significantly increased the villus height in both the jejunum and ileum, and olive oil markedly decreased the crypt depth in the jejunum and ileum. However, beef tallow (BT) markedly reduced the villus height and crypt depth in both the jejunum and ileum [3]. Dänicke et al. [16] showed that the viscosity of digestive contents could induce morphological and physiological changes in the intestine, and the inhibitory effect of BT on the development of intestinal villi attributed to a greater increase in the viscosity of jejunal and ileal contents compared with SBO. Because PF is rich in MUFA (oleic, 39.5%) and PUFA (linoleic, 23.5%) and relatively lower in SFA (palmitic, 21.4%) compared with BT and lard, PF appeared to be more efficiently utilized than other animal fats [8]. A previous study showed that supplemental PF at 0%, 5%, 10%, and 20% did not change the transition time in the gastrointestinal tract [17], which indicates that PF supplementation did not affect the viscosity of digestive contents. Therefore, in the present study, the similar viscosity of digestive contents between approximately 3% to 4% of PF supplementation and SBO supplementation may have resulted in no difference in the small intestinal morphology.

Furthermore, our results showed that PF did not affect DNA, RNA, or protein concentrations compared with SBO. Uni et al. [18] showed that RNA/DNA, RNA/protein, and protein/DNA indicated tissue activity, ribosomal capacity, and cell size, respectively. Dänicke et al. [16] showed that morphological changes in the intestine mediated by fat type might be associated with intestinal protein synthesis. In the current study, the mucosal characteristics were consistent with the intestinal weight. The results indicated that PF had no negative effect on tissue activity, ribosomal capacity, cell size, or intestinal protein synthesis compared with SBO and suggested that PF had no effect on the development of the intestinal structure at a 3% to 4% supplementation level.

Kaur et al. [19] and Ferrer et al. [4] showed that coconut oil increased the level of SFA in the brush border membrane and that corn oil increased the proportion of linoleic acid and arachidonic acid. In the current study, PF significantly decreased the content of PUFA and significantly increased the proportion of SFA and MUFA in the jejunal mucosa. This was in agreement with previous studies showing that the dietary FA composition influenced the intestinal brush border FA composition.

In the current study, PF significantly decreased FATP mRNA and L-FABP mRNA abundance at 4 or 6 wk of age compared with SBO. FATP, which is thought to be involved in both the movement of long-chain FA across the plasma membrane and esterification [20], was proposed as a major FA transporter in intestinal lipid absorption. L-FABP played a key role in transporting FA through the cytosol of absorptive cells [21]. A previous study showed that L-FABP has a higher affinity for polyunsaturated long-chain FA [22]. Oleic acid increased the L-FABP mRNA abundance in the small intestine of rats with a lower efficiency than that of linoleic acid [12]. In the current study, PF diet had a lower PUFA concentration than that of SBO, which might be the reason that PF significantly decreased FATP and L-FABP mRNA abundance. However, there was no significant difference in FATP and L-FABP mRNA abundance between PF and SBO when chickens were 2 wk of age. A previous study indicated that the ability to utilize and absorb fats, especially animal fats, was low in young poultry and was improved with increasing age; the concentrations of FABP declined from those in newly hatched chicks, but increased again after 3 wk of age, and increased significantly between 4 and 6 wk of age [23]. In our study, the lack of a difference in the abundance of L-FABP and FATP mRNA between the PF and SBO diets at 2 wk of age might have been due to the age of the chicks.

Previous studies confirmed that dietary FA composition affected the FA composition of breast muscle [24]. In the current study, our results agreed with those of Apple et al. [25], who showed that pigs fed an SBO diet had significantly reduced MUFA percentages and increased PUFA percentages and PUFA/SFA ratios compared with pigs fed a PF diet. It seemed that the dietary FA composition had similar effects on the breast muscle of both pigs and poultry. The fact that PF decreased the PUFA content in the breast muscle might be related to the lower L-FABP and FATP mRNA abundance in the jejunal mucosa compared with SBO. Because PUFA can be easily oxidated when heated, it produces various volatile compounds, including the aldehydes pentanal and hexanal, thus influencing the meat flavor [26]. In the current study, PF significantly decreased the PUFA content, especially linoleic acid and linolenic acid, the main PUFA in chicken breasts [27]. This indicates that PF might have a negative effect on the meat flavor of poultry, although this requires further confirmation.

## Conclusions

PF had no influence on intestinal development. However, PF decreased FATP and L-FABP mRNA expression in the later growth period and decreased the content of PUFA, especially linoleic acid and linolenic acid, and the PUFA/SFA ratios in breast muscle of chickens compared with SBO.

## Competing interests

The authors declare that they have no competing interests.

## Authors’ contributions

Jianmin Yuan conceived of the study, and carried out the animal trail, finished the statistical analysis and drafted the manuscript. Binkun Zhang participated in tissue sampling and molecular genetic studies. Yuming Guo was supervision of the research group, and supplied the funding. All authors read and approved the final manuscript.

## Authors’ information

Dr. Jianmin Yuan, associate professor of China Agricultural University, Focus on development of constructure and function of digestive organ, and modification of product quality in poultry.
